# Ag_2_PdP_2_O_7_

**DOI:** 10.1107/S1600536808038518

**Published:** 2008-11-29

**Authors:** Kosta Panagiotidis, Robert Glaum

**Affiliations:** a Institut für Anorganische Chemie, Rheinische Friedrich-Wilhelms-Universität Bonn, Gerhard-Domagk-Strasse 1, D-53121 Bonn, Germany

## Abstract

Disilver(I) palladium(II) diphosphate, Ag_2_PdP_2_O_7_, is isotypic with Na_2_PdP_2_O_7_. It consists of infinite diphosphato-pallad­ate(II) [Pd(P_2_O_7_)_2/2_]^2−^ ribbons with the Pd^II^ ion in an almost square-planar coordination (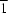 symmetry) and the P_2_O_7_ group exhibiting 2 symmetry. The [Pd(P_2_O_7_)_2/2_]^2−^ ribbons are linked by distorted [AgO_6_] octa­hedra. ^31^P-MAS NMR studies on Ag_2_PdP_2_O_7_ are in accordance with one independent site for phospho­rus; its isotropic chemical shift δ_iso_ = 21.5 p.p.m. is similar to that of Pd_2_P_2_O_7_.

## Related literature

For related literature on palladium oxo-compounds, see: Arndt & Wickleder (2007 ▶); Dahmen *et al.* (1994 ▶); Laligant *et al.* (1991 ▶); Palkina *et al.* (1978 ▶); Panagiotidis *et al.* (2005*b* ▶); Waser *et al.* (1953 ▶). For related literature on polynary palladium phosphates, see: El Maadi *et al.* (2003 ▶); Laligant (1992*a* ▶,*b* ▶); Lii *et al.* (2004 ▶); For related literature on noble metal phosphates, see: Panagiotidis *et al.* (2005*a* ▶, 2008 ▶). For background on chemical shift parameters, see: Moreno *et al.* (2002 ▶); Griffiths *et al.* (1986 ▶); Hayashi & Hayamizu (1989 ▶). For details of software used, see: Bak *et al.* (2000 ▶); Soose & Meyer (1980 ▶); Vosegaard *et al.* (2002 ▶).

## Experimental

### 

#### Crystal data

Ag_2_PdP_2_O_7_
*M* *_r_* = 496.10
Monoclinic, 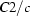

*a* = 15.739 (2) Å
*b* = 5.7177 (7) Å
*c* = 8.187 (1) Å
β = 116.75 (1)°
*V* = 657.91 (15) Å^3^
*Z* = 4
Mo *K*α radiation
μ = 9.08 mm^−1^
*T* = 293 (2) K
0.08 × 0.05 × 0.05 mm

#### Data collection

Enraf–Nonius CAD-4 diffractometer
Absorption correction: ψ scan (North *et al.*, 1968 ▶) *T*_min_ = 0.551, *T*_max_ = 0.631
1890 measured reflections
947 independent reflections
591 reflections with *I* > 2σ(*I*)
*R*_int_ = 0.080
3 standard reflections frequency: 60 min intensity decay: none

#### Refinement

*R*[*F* ^2^ > 2σ(*F* ^2^)] = 0.035
*wR*(*F* ^2^) = 0.074
*S* = 0.97
947 reflections
58 parameters
Δρ_max_ = 1.30 e Å^−3^
Δρ_min_ = −1.14 e Å^−3^

### 

Data collection: *CAD-4 EXPRESS* (Enraf–Nonius, 1994 ▶); cell refinement: *CAD-4 EXPRESS*; data reduction: *XCAD4* (Harms & Wocadlo, 1995 ▶); program(s) used to solve structure: *SHELXS97* (Sheldrick, 2008 ▶); program(s) used to refine structure: *SHELXL97* (Sheldrick, 2008 ▶); molecular graphics: *DIAMOND* (Brandenburg, 2008 ▶); software used to prepare material for publication: *WinGX* (Farrugia, 1999 ▶).

## Supplementary Material

Crystal structure: contains datablocks I, global. DOI: 10.1107/S1600536808038518/br2086sup1.cif

Structure factors: contains datablocks I. DOI: 10.1107/S1600536808038518/br2086Isup2.hkl

Additional supplementary materials:  crystallographic information; 3D view; checkCIF report

## Figures and Tables

**Fig. 1.**
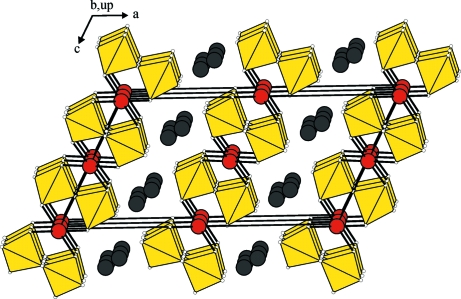
Projection of the crystal structure of Ag2PdP2O7 along [010] with tetrahedral [PO4] units (yellow), Pd2+ (red) and Ag+ grey (DIAMOND v3.1f).

**Fig. 2.**
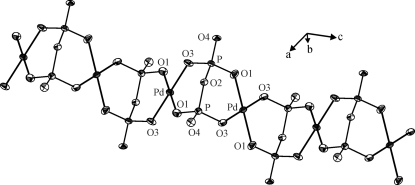
Diphosphato-palladate(II) ribbon [Pd(P2O7)2/2]2- along [001]. Thermal elipsoids with 50% probability (DIAMOND, v3.1f).

## supplementary crystallographic information

### Comment

With the synthesis and crystal structure refinement of the first gold phosphate Au^III^PO_4_ (Panagiotidis *et al.*, 2005*a*) and two modifications of Ir^III^(PO_3_)_3_ (Panagiotidis *et al.*, 2008) we have widened the crystal chemical knowledge on anhydrous phosphates of the noble metals. Investigations in the ternary system Pd/P/O provided, apart from the already existing phosphates Pd(PO_3_)_2_ (Palkina *et al.*, 1978) and Pd_2_P_2_O_7_ (Panagiotidis *et al.*, 2005*b*), no evidence for further thermodynamically stable palladium phosphates. Due to our interest in network structures built from square-planar units [*M*O_4_] (*M* = Pd^II^, Au^III^) and phosphate tetrahedra we focused therefore our search on polynary palladium phosphates. Polynary phosphates of divalent palladium are rare in literature. Up to now, only the compositions *M*^I^_2_PdP_2_O_7_ (*M* = Li (Laligant, 1992*a*), Na (Laligant, 1992*b*), K (El Maadi *et al.*, 2003), K_3.5_Pd_2.25_(P_2_O_7_)_2_ (El Maadi *et al.*, 2003) and Cs_2_Pd_3_(P_2_O_7_)_2_ (Lii *et al.*, 2004) were reported. In Pd_2_P_2_O_7_ itself, Li_2_PdP_2_O_7_, and Na_2_PdP_2_O_7_ infinite ribbons [Pd(P_2_O_7_)_2/2_]^2-^ are the characteristic structural motif. K_2_PdP_2_O_7_ adopts a layer structure with the crystal chemical composition [Pd(P_2_O_7_)_4/4_]^2-^. The structures of K_3.5_Pd_2.25_(P_2_O_7_)_2_ and Cs_2_Pd_3_(P_2_O_7_)_2_ consist of [Pd^II^O_4_] and [P_2_O_7_] groups generating a three-dimensional framework.

According to our X-ray single-crystal study Ag_2_PdP_2_O_7_ is isotypic to Na_2_PdP_2_O_7_. The unit cell contains four formula units Ag_2_PdP_2_O_7_ with one crystallographically independent site for silver, palladium and phosphorus (Fig. 1). As in the crystal structures of PdO (Waser *et al.*, 1953), *M*-Pd^II^SO_4_ (Dahmen *et al.*, 1994), Pd^II^(NO_3_)_2_(H_2_O)_2_ (Laligant *et al.*, 1991), and Pd_2_P_2_O_7_ the Pd^2+^ ions show a square-planar coordination by oxygen. In Ag_2_PdP_2_O_7_ palladium is coordinated in a chelating way by two [P_2_O_7_] groups. This coordination mode, with a, for such diphosphates typically observed, bridging angle 〈(P—O2—P) = 124.9°, leads to the formation of corrugated ribbons [Pd(P_2_O_7_)_2/2_]^2-^ (Fig. 2). These ribbons are linked by significantly distorted [Ag^I^O_6_] octahedra. Due to different crystal chemical environment of the four independent oxygen atoms, with O1 forming one bond to P and two to Ag, O2 forming two bonds to P and O3 and O4 forming one bond each to P, Pd and Ag, a radial distortion of the phosphate groups with one very short, two medium long and one elongated distance d(P—O) is observed. In accordance with the crystal structure of Ag_2_PdP_2_O_7_^31^P-MAS-NMR investigations (Varian Infinity Plus, 9.4 tesla-magnet, 2.5-mm MAS double resonance NMR probe, rotation frequency 3.0 kHz) show the presence of just one phosphorus site. Chemical shift parameters were determined by means of numerically calculated spectra (programme SIMPSON (Bak *et al.*, 2000), *MINUIT* routine in SIMPSON (Vosegaard *et al.*, 2002)) to δ_iso_ = 21.5 p.p.m., δ_aniso_ = 79.0 p.p.m. and η = 0.87. The chemical shifts are reported in parts per million (p.p.m.) from the external standard 85% H_3_PO_4_. As in Pd_2_P_2_O_7_ (η = 0.86) and in contrast to other diphosphates (Moreno *et al.*, 2002; Griffiths *et al.*, 1986; Hayashi & Hayamizu, 1989) a remarkably high value for η is observed. The isotropical chemical shift of Ag_2_PdP_2_O_7_ which is similar to the one observed for Pd_2_P_2_O_7_ (δ_iso_ = 28.3 p.p.m.) is exceptionally high in comparison to δ_iso_ values of diphosphates of the alkaline and alkaline earth metals (Moreno *et al.*, 2002; Griffiths *et al.*, 1986; Hayashi & Hayamizu, 1989). We attribute this observation to significant covalency in the Pd—O interaction.

### Experimental

Microcrystalline Ag_2_PdP_2_O_7_ was synthesized *via* a solid state reaction by heating an amorphous precursor for 24 h at T = 773 K in air. The precursor was obtained by drying a mixture of 100.0 mg (0.94 mmol) palladium powder (99.99%, UMICORE AG, Hanau–Wolfgang) with an excess of conc. nitric acid and stoichiometric amounts of 319.2 mg AgNO_3_ (1.88 mmol) (p.A., Merck) and 18.8 ml H_3_PO_4_ (0.1 *M*) at 423 K as a brownish powder.

Isothermal heating of 100.0 mg (0.82 mmol) PdO, 189.3 mg (0.82 mmol) Ag_2_O (p.A. Merck) and 116.0 mg (0.41 mmol) P_4_O_10_ (99%, Riedel de Häen) (addition of 8.0 mg PdCl_2_ as mineralizer) carried out in sealed and evacuated silica tubes at 773 K for seven days gave besides microcrystalline, single-phase Ag_2_PdP_2_O_7_ (eq. 1) also small amounts of yellow plate-like single crystals which were distributed over the whole ampoule.

PdO_s_ + Ag_2_O_s_ + 1/2 P_4_O_10,s_→ Ag_2_PdP_2_O_7,s_ (eq. 1)

### Crystal data

**Table:**

Ag_2_PdP_2_O_7_	The lattice parameters given were refined with the program SOS (Soose & Meyer, 1980), using 40 reflections from a Guinier IP photograph.
*M**_r_* = 496.10	*D*_x_ = 5.008 Mg m^−^^3^
Monoclinic, *C*2/*c*	Mo *K*α radiation λ = 0.71073 Å
Hall symbol: -C 2yc	Cell parameters from 40 reflections
*a* = 15.739 (2) Å	θ = 6.3–34.3º
*b* = 5.7177 (7) Å	µ = 9.08 mm^−^^1^
*c* = 8.187 (1) Å	*T* = 293 (2) K
β = 116.75 (1)º	Cell measurement pressure: 101.3 kPa
*V* = 657.91 (15) Å^3^	Prism, yellow
*Z* = 4	0.08 × 0.05 × 0.05 mm
*F*_000_ = 904	

### Data collection

**Table:**

Enraf–Nonius CAD-4 diffractometer	591 reflections with *I* > 2σ(*I*)
Radiation source: fine-focus sealed tube	*R*_int_ = 0.080
Monochromator: graphite	θ_max_ = 29.9º
*T* = 293(2) K	θ_min_ = 2.9º
*P* = 101.3 kPa	*h* = −22→22
Nonprofiled ω scans	*k* = −8→0
Absorption correction: ψ scan(North *et al.*, 1968)	*l* = −11→11
*T*_min_ = 0.551, *T*_max_ = 0.631	3 standard reflections
1890 measured reflections	every 60 min
947 independent reflections	intensity decay: none

### Refinement

**Table:**

Refinement on *F*^2^	Secondary atom site location: difference Fourier map
Least-squares matrix: full	*w* = 1/[σ^2^(*F*_o_^2^) + (0.0245*P*)^2^] where *P* = (*F*_o_^2^ + 2*F*_c_^2^)/3
*R*[*F*^2^ > 2σ(*F*^2^)] = 0.035	(Δ/σ)_max_ < 0.001
*wR*(*F*^2^) = 0.074	Δρ_max_ = 1.30 e Å^−^^3^
*S* = 0.97	Δρ_min_ = −1.14 e Å^−^^3^
947 reflections	Extinction correction: SHELXL97 (Sheldrick, 2008), Fc^*^=kFc[1+0.001xFc^2^λ^3^/sin(2θ)]^-1/4^
58 parameters	Extinction coefficient: 0.00104 (17)
Primary atom site location: structure-invariant direct methods	

### Special details

**Table:**

Geometry. All e.s.d.'s are estimated using the full covariance matrix. The cell e.s.d.'s are taken into account individually in the estimation of e.s.d.'s in distances and angles; correlations between e.s.d.'s in cell parameters are only used when they are defined by crystal symmetry.
Refinement. Refinement of *F*^2^ against ALL reflections. The weighted *R*-factor *wR* and goodness of fit *S* are based on *F*^2^, conventional *R*-factors *R* are based on *F*, with *F* set to zero for negative *F*^2^. The threshold expression of *F*^2^ > σ(*F*^2^) is used only for calculating *R*-factors(gt) *etc*. and is not relevant to the choice of reflections for refinement. *R*-factors based on *F*^2^ are statistically about twice as large as those based on *F*, and *R*- factors based on ALL data will be even larger.

### Fractional atomic coordinates and isotropic or equivalent isotropic displacement parameters (Å^2^)

**Table:**

	*x*	*y*	*z*	*U*_iso_*/*U*_eq_	
Pd1	0	0	0	0.0145 (2)	
Ag1	0.23426 (5)	0.85894 (13)	0.79398 (9)	0.0217 (2)	
P1	0.10116 (14)	0.3445 (4)	0.8422 (3)	0.0137 (4)	
O1	0.8200 (4)	0.5226 (10)	0.5959 (7)	0.0177 (13)	
O2	0	0.4744 (14)	0.75	0.0139 (16)	
O3	0.8949 (4)	0.1859 (11)	0.8057 (7)	0.0220 (14)	
O4	0.6031 (4)	0.2953 (11)	0.5045 (8)	0.0213 (14)	

### Atomic displacement parameters (Å^2^)

**Table:**

	*U*^11^	*U*^22^	*U*^33^	*U*^12^	*U*^13^	*U*^23^
Pd1	0.0103 (4)	0.0178 (5)	0.0135 (4)	−0.0012 (4)	0.0036 (3)	0.0055 (4)
Ag1	0.0197 (4)	0.0247 (4)	0.0171 (3)	−0.0044 (3)	0.0050 (3)	−0.0017 (3)
P1	0.0111 (9)	0.0152 (11)	0.0146 (9)	−0.0028 (9)	0.0055 (8)	0.0003 (10)
O1	0.016 (3)	0.016 (3)	0.019 (3)	0.011 (3)	0.006 (2)	0.005 (3)
O2	0.014 (4)	0.011 (4)	0.015 (4)	0	0.006 (3)	0
O3	0.018 (3)	0.027 (4)	0.019 (3)	−0.003 (3)	0.007 (3)	0.010 (3)
O4	0.011 (3)	0.026 (4)	0.023 (3)	0.004 (3)	0.004 (2)	−0.012 (3)

### Geometric parameters (Å, °)

**Table:**

Pd1—O4^i^	1.987 (5)	P1—O4^x^	1.539 (6)
Pd1—O4^ii^	1.987 (5)	P1—O2	1.605 (4)
Pd1—O3^iii^	2.007 (6)	O1—P1^vii^	1.505 (6)
Pd1—O3^iv^	2.007 (6)	O1—Ag1^xi^	2.321 (5)
Ag1—O1^v^	2.321 (5)	O1—Ag1^vii^	2.436 (6)
Ag1—O4^vi^	2.368 (6)	O2—P1^xii^	1.605 (4)
Ag1—O1^vii^	2.436 (6)	O3—P1^vii^	1.537 (6)
Ag1—Ag1^viii^	3.0427 (6)	O3—Pd1^xiii^	2.007 (6)
Ag1—Ag1^ix^	3.0427 (6)	O4—P1^xiv^	1.539 (6)
P1—O1^vii^	1.505 (6)	O4—Pd1^xv^	1.987 (5)
P1—O3^vii^	1.537 (6)	O4—Ag1^xvi^	2.368 (5)
			
O4^i^—Pd1—O4^ii^	180.0 (4)	Ag1^viii^—Ag1—Ag1^ix^	139.96 (5)
O4^i^—Pd1—O3^iii^	94.5 (2)	O1^vii^—P1—O3^vii^	110.2 (3)
O4^ii^—Pd1—O3^iii^	85.5 (2)	O1^vii^—P1—O4^x^	111.6 (3)
O4^i^—Pd1—O3^iv^	85.5 (2)	O3^vii^—P1—O4^x^	112.4 (4)
O4^ii^—Pd1—O3^iv^	94.5 (2)	O1^vii^—P1—O2	109.8 (4)
O3^iii^—Pd1—O3^iv^	180.0 (6)	O3^vii^—P1—O2	106.6 (3)
O1^v^—Ag1—O4^vi^	159.7 (2)	O4^x^—P1—O2	106.0 (3)
O1^v^—Ag1—O1^vii^	88.23 (19)	P1^vii^—O1—Ag1^xi^	123.5 (3)
O4^vi^—Ag1—O1^vii^	87.5 (2)	P1^vii^—O1—Ag1^vii^	141.5 (3)
O1^v^—Ag1—Ag1^viii^	116.60 (15)	Ag1^xi^—O1—Ag1^vii^	91.77 (19)
O4^vi^—Ag1—Ag1^viii^	77.30 (15)	P1^xii^—O2—P1	124.9 (5)
O1^vii^—Ag1—Ag1^viii^	57.82 (14)	P1^vii^—O3—Pd1^xiii^	128.7 (3)
O1^v^—Ag1—Ag1^ix^	84.21 (15)	P1^xiv^—O4—Pd1^xv^	126.2 (3)
O4^vi^—Ag1—Ag1^ix^	93.96 (16)	P1^xiv^—O4—Ag1^xvi^	127.8 (3)
O1^vii^—Ag1—Ag1^ix^	161.97 (14)	Pd1^xv^—O4—Ag1^xvi^	105.4 (2)

Symmetry codes: (i) *x*−1/2, −*y*+1/2, *z*−1/2; (ii) −*x*+1/2, *y*−1/2, −*z*+1/2; (iii) *x*−1, *y*, *z*−1; (iv) −*x*+1, −*y*, −*z*+1; (v) *x*−1/2, −*y*+3/2, *z*+1/2; (vi) *x*−1/2, *y*+1/2, *z*; (vii) −*x*+1, *y*, −*z*+3/2; (viii) −*x*+1/2, *y*−1/2, −*z*+3/2; (ix) −*x*+1/2, *y*+1/2, −*z*+3/2; (x) *x*−1/2, −*y*+1/2, *z*+1/2; (xi) *x*+1/2, −*y*+3/2, *z*−1/2; (xii) −*x*, *y*, −*z*+3/2; (xiii) *x*+1, *y*, *z*+1; (xiv) *x*+1/2, −*y*+1/2, *z*−1/2; (xv) −*x*+1/2, *y*+1/2, −*z*+1/2; (xvi) *x*+1/2, *y*−1/2, *z*.
